# Developing a stress induction tool relevant to relationships in a health context

**DOI:** 10.3389/fpsyg.2023.1103081

**Published:** 2023-02-09

**Authors:** Youngmee Kim, Charles S. Carver, Barry E. Hurwitz

**Affiliations:** Department of Psychology, University of Miami, Coral Gables, FL, United States

**Keywords:** stress induction tool, close relationships, health, experimental design, cardiovascular reactivity, negative affect, individual differences

## Abstract

Concerns pertaining to health and to problems in close relationships are both known to be major stressors, yet existing tools are inadequate to assess individual reactions to such stressors. Thus, we sought to develop and preliminarily validate a stress-inducing task for use in a laboratory setting that pertains to the sorts of health-related concerns people face in close relationships. Heterosexual dating couples (44 individuals: mean age 22) were randomized to be paired with their own partner or a stranger and to play a role of speaker or listener. Participants were asked to imagine a scenario in which one person is hit by a car (listener role) and the partner has no means to provide or seek out help for the victim (speaker role). The session consisted of baseline, speech preparation, stress task, and recovery phases. General linear modeling results revealed that the task induced stress, evidenced in cardiovascular activities and self-reported negative affect. Giving a brief speech about the stressful situation creates physiological and psychological strains, regardless of pairing with one’s own partner or stranger. Furthermore, cardiovascular and negative affect reactivity to the STress Induction Tool for Close relationships and Health (STITCH) task tended to vary by individual characteristics that reflect one’s sensitivity to close relationship-and health-related stress. This tool is intended to be used for testing relationship theory-driven phenomenon and longer-term implications of physiological and affective reactivities in the quality of life and health outcomes of those who experienced a medically stressful circumstance personally or in the family.

## Introduction

Responses to acute laboratory stressors (reactivity and recovery) have long been recognized as reliable markers of variation in development of diseases even years later (Dickerson and Kemeny, 2004; Steptoe et al., 2007; Chida and Hamer, 2008; Pattyn et al., 2010; Dias and Neto, 2016; Ford et al., 2018; Giannakakis et al., 2019). One of the most widely used tasks to induce stress in the laboratory is the Trier Social Stress Task (TSST; Kirschbaum et al., 2004). The TSST involves delivering a speech and performing mental arithmetic in front of an audience or a video camera recording the performance to be evaluated by others. These procedures thus induce social evaluative stress, in the form of judgmental other people (experimenter or audience).

Although such tasks are successful stress inducers, they have important limitations. For example, the TSST provokes one particular sort of stress: social evaluative stress. Although social evaluation is an important source of stress, it differs from many other common kinds of stress that people experience in their lives, such as problems in close relationships and confrontations with medical illnesses. The TSST is also designed to create a stress response in one person at a time. However, many kinds of stress involve more than one person who mutually experience the common stressor, such as concerns pertaining to health and problems in close relationships. Both kinds of stress are known to be major stressors (Slavich, 2016), which have been significant predictors of adverse neuroendocrine and immune responses, poor quality of life, and even the developing of major diseases (Dickerson and Kemeny, 2004; Robles et al., 2014). If each of these two kinds of stressors is problematic in itself, joining them to one another is likely to exacerbate the adverse effect of each. How people react to these sorts of stressors is likely not well captured by the TSST or other existing commonly used lab stressors.

Further, in line with the adult attachment theory (Hazan and Shaver, 1987; Feeney and Kirkpatrick, 1996; Fraley and Shaver, 2000) and the social baseline theory (Beckes and Coan, 2011), adult family relationships are an interdependent system, in which stress and its regulation by one person involves not only that person’s own experience but also that of the partner. Thus, for example, a new medical illness for one family member is a stressor shared by all adult family members. The diagnosis imposes additional and mutual challenges on the relationship, with the introduction of new roles of patient and caregiver. Major medical illnesses also impose repeated or cyclic challenges over a long period of time.

Common stressors for patients with a medical illness include dealing with the disease itself and also feeling that their illness (and they themselves) burden their family members in numerous ways (Hagger and Orbell, 2003; Kim et al., 2016). Common stressors for caregivers include attempting to minimize the patient’s suffering and providing diverse types of support to the patient, and carrying out their own existing social roles and roles that have been newly added by the patient’s illness, all while managing their own emotional upheaval brought on by the patient’s illness (Kim and Given, 2008; Northouse et al., 2010; Kent et al., 2016). In addition to attachment relationship orientations, individual differences in stress perception and regulation, such as personality traits, have also been associated with different adjustment outcomes (McCrae et al., 2005).

An important goal is understanding how the shared stress and stress regulation that follow from a serious medical diagnosis affects the mental and physical health of each person in the family, ultimately resulting in long-term poor quality of life and development of morbidities. How to study the stress regulation of families confronting illness presents a real challenge, however. The time surrounding a diagnosis involves demands at an individual, family, and medical system level. There are multiple difficulties in obtaining information about the family’s reactions to these demands. It often is difficult to have patients and family members participate in psychosocial research during the period of a medical crisis, and retrospective information about such experiences is subject to substantial recall bias.

Thus, we sought to develop and validate a stress-inducing task that is pertinent to the sorts of health-related concerns people face in close relationships. In essence, we sought to create an analog of illness-related relationship stress that incorporated a challenge to physical health in a laboratory setting. The task we developed used a standardized event—a hypothetical accident in which one person (representing the patient) is badly injured by an impaired driver who fled the scene, while the partner (representing the caregiver) is left otherwise alone and helpless. Dyads were asked to imagine themselves in this situation as vividly as possible and consider the thoughts and feelings they would have. The caregiver then spoke about those thoughts and feelings while the patient listened.

Using a laboratory analog in which the same context is presented to everyone, rather than trying to assess stress regulation in the medical setting itself has several benefits. First, as noted above, there are both practical and ethical issues involved in trying to assess these responses in real time during the medical crisis itself. Instead, stress regulation between a dyad can be assessed later on, at a time that does not intrude on the medical event. Second, using a standardized event for the lab task reduces variability between individuals and between couples stemming from exposure to different medical systems, different actual medial events, and so on. Standardizing the requirements of the task, and using a context that, although health-related, is not the same medical situation as the patient actually has been dealing with will reduce effects of variations in wishing not to talk about the patient’s medical illness itself while “moving on” after the initial medical treatment.

In the work reported here, the newly developed task in this study was validated by assessment of cardiovascular activity and self-reported negative affect. Cardiovascular activity indicators and self-reported negative affect across various stress induction phases and study conditions were chosen as primary outcomes, as the link from conflict in close relationships to these indicators has been well-documented (Nealey-Moore et al., 2007; Chida and Steptoe, 2010). We hypothesized that cardiovascular activity indicators and self-reported negative affect would peak when the task stress is induced, and that it would be more prominent for the person delivering a speech about the stress experience (as opposed to listening to the speech). We also expected that stress would be greater when the person was paired in the stressful task with his or her own romantic partner (as opposed to being paired with a stranger). For this purpose, we decided to study young dating couples to randomly assign them to either pairing condition, as opposed to old cancer patient-caregiver dyads.

A second approach to validation was to examine individual difference characteristics that are sensitive to close relationship-and health-related stressors. We hypothesized that attachment anxiety and neuroticism would be related to greater cardiovascular activity and negative affect at baseline, reflecting the trait characteristics of hyperarousal to relationship loss or distress. Attachment anxiety and agreeableness were also hypothesized to be sensitive to the onset of the stressor reflecting a threat to close relationship and health (Kim, 2006; Dimsdale, 2008; Schneider et al., 2012; Pietromonaco and Beck, 2019); thus its association with outcomes would vary significantly across study phases.

## Methods

### Participants

Young adult couples were recruited for this study from the university campus using flyers and by word of mouth. Eligibility criteria were to be 18 years or older and to have been in a committed relationship for at least 3 months with a heterosexual partner at the time of participation. Individuals participated in the study for course credits or for cash in response to study flyers. A total of 44 individuals (22 couples) participated in this study.

### Procedure

This study was conducted in compliance with the regulations of the University of Miami Institutional Review Board from February to May 2011. No foods or drinks were allowed for 30 min before arrival to the laboratory. Upon arrival, participants provided informed consent individually. Next, participants were assigned to one of the two study conditions: paired with their own romantic partner or with a stranger. The pair then moved to an experimental room, where a blood pressure cuff and three electrodes were placed on each participant before he/she began filling out questionnaires for the next 30 min, allowing blood pressure to reach a baseline (Baseline Phase).

At the end of the baseline phase, individuals within a dyad were randomly assigned to one of two experimental roles, speaker or listener, in a scenario that was to be read aloud by the experimenter. An eight-minute stress period (3 min of Preparation Phase and 5 min of Stress Task Phase) was followed by 22 min of Recovery. During the recovery phase, participants continued filling out questionnaires with no further tasks. Finally, participants were debriefed about the study and given appropriate incentive of either course credits or having the option to either receive a $30 check or enter their names into a $200 raffle, as a couple. Participants were given an opportunity to revoke their consent to participate in the study after the debriefing.

### Stress task

The STress Induction Tool for Close relationships and Health (STITCH) task was developed for this study in order to induce stress that is pertinent to close relationships, and to do so in the context of health issues. The STITCH requires the dyad to imagine being involved in a car accident in which one is hit by a drunk driver who drives away, leaving the partner helpless in the middle of the road late at night in an unfamiliar neighborhood without convenient access to immediate help (see Appendix for full STITCH scenario).

The person randomized to the speaker role at the beginning of the preparation phase was instructed to identify him/herself with the character in the scenario whose partner was injured, as a proxy of caregiver; the listener was instructed to identify him/herself with the character in the scenario who had been injured, as a proxy of the patient. The experimenter then read the scenario to the couple. After that, the speaker was given 3 min to prepare for a speech describing as vividly as possible how he/she would feel physically and emotionally and what he/she might attempt to do if he/she was in the situation described in the scenario. The listener was instructed writing a paragraph describing the classroom of a class he/she took last week, not to engage in any interaction with the speaker, and also to simulate the unconscious condition of the victim. After the preparation phase, the speaker spoke for 5 min. If he/she stopped before the end of 5 min, the experimenter instructed the speaker to reiterate what had been said. While the speaker presented his/her speech, the listener was asked to simply sit still and listen to the speaker.

### Measures

#### Cardiovascular activity

Each individual’s systolic blood pressure (SBP, mmHg), diastolic blood pressure (DBP, mmHg), and heart rate (HR, beats per min) were measured using a Critikon Dinamap (model 1846SX) Adult/Pediatric Vital Signs Monitor. The occlusion cuff was placed on the upper portion of the arm. The three indicators of cardiovascular activity were measured at approximately 1.5 min intervals during a given study phase. The two final baseline recordings were used to represent baseline value before the stress task was introduced. In the preparation phase, recordings were made at 1.5 and 3 min (end of that phase). In the 5 min of the Stress Task Phase, recordings were made at 1.5, 3, and 4.5 min. At the start of the recovery phase, three recordings were obtained at 1.5, 3, and 4.5 min, to capture the initial recovery patterns immediately after removal of the stress. The values of each cardiovascular activity indicator within a phase were averaged. Data for each measure thus consisted of one mean for each of the study phases: baseline, preparation, stress task, and recovery.

#### Negative affect

Each individual’s perceived stress at the moment was measured using three adjectives (stressful, unpleasant, and strained) responding to a question “how do you feel right now?” on a 5-point Likert type scale (1 = not at all; 5 = very much) at the end of each phase. The three items were averaged: higher scores indicating greater self-reported negative affect. Internal consistency during the stress induction phases (preparation, stress task, and recovery phases) was good (αs = 0.85, 0.83, and 0.72, respectively), while that before the stress induction was acceptable (α = 0.59).

#### Adult attachment

The qualities of attachment that participants felt with respect to their romantic partner were measured dimensionally, using the Measure of Attachment Qualities, or MAQ (Carver, 1997). MAQ items are statements, answered for extent of agreement on a 4-point Likert-type scale (1 = *strongly disagree*, 4 = *strongly agree*). The MAQ has four subscales, one reflecting security (e.g., “It feels relaxing and good to be close to him/her”), one reflecting anxiety related to worry (e.g., “I often worry that he/she does not really love me”), one reflecting anxiety related to desire to merge (e.g., “I have trouble getting others to be as close as I want them to be”), and one reflecting avoidance (e.g., “I prefer not to be too close to him/her”). Each of the four sub-scales had adequate internal consistency: security (3 items, α = 0.69); anxiety-worry (3 items, α = 0.69); anxiety-merger (3 items, α = 0.74); and avoidance (5 items, α = 0.72). Each sub-scale was scored by averaging responses (after appropriate reversals). Security was inversely and fairly substantially related to avoidance, *r* = −0.62, *p* < 0.001, but was not significantly related to anxiety-worry and anxiety-merger (*r*s < −0.18, *p*s > 0.22); correlations of anxiety-worry with anxiety-merger and avoidance were positive and significant (*r* = 0.502, *p* < 0.001; *r* = 0.374, *p* = 0.017, respectively); and correlation of anxiety-merger with avoidance was 0.43, *p* < 0.006.

#### Big five personality

Individual differences in the five major personality factors (extraversion, agreeableness, conscientiousness, neuroticism, and agreeableness) were assessed using the 25-item NEO-FFI (Costa and McCrae, 1992; McCrae et al., 2005) on a 5-point Likert-type scale. Each factor was scored by averaging corresponding items (after appropriate reversals). Each of the five factors (5 items per factor) had adequate internal consistency (0.64 < αs < 0.81) and represented fairly distinct characteristics to each other (|0.002| < *r*s < |0.307|, 0.057 < *p*s < 0.992).

### Analytic plans

Mean, standard deviation, and frequency of study variables were computed. The primary aim was to validate the stress-inducing task, STITCH, in two ways. First, whether cardiovascular activities and negative affect changed in response to the STITCH task was examined using general linear modeling. Each of the cardiovascular activity and negative affect markers was predicted independently by study phases (repeated measures), experimental role (speaker vs. listener), experimental condition (paired with own partner or a stranger), and the two-way interaction effects with study phases.

The validity of the STITCH task with regard to inducing stress would be supported by the following effects. A significant curvilinear (inverse U-shape with peaks during preparation and stress task phases) time effect would indicate an overall stress response. The interaction of time with experimental role (speaker showing greater stress reaction) would support the predicted role difference. And the interaction of time with experimental condition predicted experimental condition difference.

Regarding the validity of the STITCH task by individual difference characteristics would be supported by attachment anxiety and neuroticism associated with greater cardiovascular activity and negative affect at baseline; and attachment anxiety and agreeableness associated with greater variability in outcomes across study phases. Significance level in all analyses was set at *p* < 0.05. Significance at *p* < 0.10 was interpreted with caution.

## Results

### STITCH task validation with cardiovascular activity and negative affect

Study participants were primarily either non-Hispanic or Hispanic White young adults. Their cardiovascular activity indicators were normative for their age during the resting baseline phase (Tables 1, 2). As shown in Table 2, curvilinear time effects, showing increases from baseline to preparation and stress task periods and decreases afterward, of all three cardiovascular activity indicators and negative affect were significant (*p*s < 0.03). Both SBP and DBP increased to peak at the stress task phase and then subsided at the recovery phase. DBP value at the recovery phase completely returned to the initial resting baseline level [paired *t*-tests between baseline and recovery values: *t*_(1,43)_ = 1.05, *p* = 0.30]; that of SBP remained marginally elevated [paired *t*_(1,43)_ = 1.86, *p* = 0.069]. HR peaked equally at the preparation and stress task phases then subsided at the recovery phase, but did not return to baseline [paired *t*_(1,43)_ = 2.04, *p* = 0.047]. Self-reported negative affect increased to peak at the preparation phase and then subsided by the end of the recovery phase to be similar to the initial baseline level [paired test between baseline and recovery *t*_(1,43)_ = 0.53, *p* = 0.60].

**Table 1:** Sample characteristics and descriptive statistics for study variables.

	Mean (SD) or %	*N*
Age	21.51 (2.35)	40
Gender (female)	50%	44
Ethnicity: Non-hispanic white	43.2%	19
Hispanic	40.9%	18
African American	9.1%	4
Other	6.8%	3
**Attachment**		
Security	3.72 (0.37)	40
Avoidant	1.32 (0.43)	40
Anxiety-worry	1.58 (0.61)	40
Anxiety-merger	1.51 (0.60)	40
**Big five personality**		
Extraversion	3.26 (0.76)	39
Agreeableness	4.27 (0.51)	39
Conscientiousness	3.93 (0.76)	39
Neuroticism	2.41 (0.86)	39
Openness	3.58 (0.71)	39
**Baseline cardiovascular activity**		
SBP	110.98 (9.23)	44
DBP	66.36 (6.00)	44
HR	66.81 (9.48)	44
Baseline negative affect	1.36 (0.42)	44

**Table 2:** Descriptives of cardiovascular activity indicators and negative affect by study phases, experimental role (speaker vs. listener), and experimental condition (paired with own partner: couple vs. with stranger); and *t*-test for each role or condition effects within a study phase.

	Study phases								Time and time by role or condition effects					
	Baseline		Preparation		Stress task		Recovery		Linear		Quadratic		Cubic	
	*M*	*SD*	*M*	*SD*	*M*	*SD*	*M*	*SD*	*F*	*p*	*F*	*p*	*F*	*p*
SBP	110.98	(9.23)	117.82	(14.94)	120.70	(16.55)	114.39	(12.13)	10.71	0.002	20.14	0.001	5.18	0.028
Speaker	111.57	(9.04)	122.61	(14.57)	128.00	(17.27)	117.68	(12.67)	8.13	0.007	9.41	0.004	4.80	0.034
Listener	110.39	(9.60)	113.02	(14.02)	113.40	(12.30)	111.11	(10.86)						
*t*	0.42		2.23^*^		3.23^**^		1.85							
Couple	111.17	(9.09)	119.73	(14.44)	123.23	(15.67)	114.69	(11.78)	1.08	0.784	2.74	0.106	0.85	0.362
Stranger	110.69	(9.70)	115.06	(15.63)	117.04	(17.54)	113.96	(12.94)						
*t*	−0.17		−1.02		−1.23		−0.19							
DBP	66.36	(6.00)	71.10	(8.16)	73.20	(9.28)	67.45	(6.89)	5.90	0.019	54.54	0.001	11.46	0.002
Speaker	68.45	(5.99)	74.84	(6.68)	79.14	(6.77)	70.00	(6.33)	2.78	0.103	17.41	0.001	25.12	0.001
Listener	64.27	(5.36)	67.36	(7.90)	67.25	(7.50)	64.89	(6.58)						
*t*	2.44^*^		3.39^**^		5.52^***^		2.62^*^							
Couple	67.69	(5.88)	73.00	(7.99)	75.11	(9.10)	67.78	(7.68)	2.74	0.105	3.48	0.069	0.64	0.428
Stranger	64.44	(5.81)	68.36	(7.99)	70.44	(9.07)	66.96	(5.72)						
*t*	−1.81^†^		−1.91^†^		−1.67		−0.38							
HR	66.81	(9.48)	76.58	(14.83)	76.01	(14.21)	69.74	(9.53)	11.16	0.002	30.30	0.001	9.72	0.003
Speaker	67.70	(11.65)	79.80	(19.29)	80.02	(18.08)	69.38	(11.02)	0.12	0.729	7.42	0.009	4.22	0.046
Listener	66.61	(6.94)	73.36	(7.49)	71.99	(7.25)	70.11	(8.02)						
*t*	0.13		1.46		1.93^†^		−0.25							
Couple	66.31	(10.03)	76.71	(17.09)	75.72	(16.87)	68.09	(9.43)	3.81	0.058	0.68	0.416	0.01	0.940
Stranger	67.53	(8.86)	76.39	(11.25)	76.42	(9.57)	72.13	(9.42)						
*t*	0.42		−0.07		0.16		1.40							
NA	1.36	(0.42)	2.46	(1.03)	1.52	(0.64)	1.40	(0.50)	7.62	0.008	37.48	0.001	57.16	0.001
Speaker	1.29	(0.40)	2.95	(0.99)	1.64	(0.78)	1.41	(0.54)	0.24	0.630	15.25	0.001	12.24	0.001
Listener	1.42	(0.43)	1.97	(0.83)	1.39	(0.43)	1.39	(0.46)						
*t*	−1.09		3.57^***^		1.27		0.10							
Couple	1.32	(0.38)	2.28	(1.13)	1.50	(0.76)	1.38	(0.53)	0.81	0.372	0.74	0.395	2.32	0.136
Stranger	1.41	(0.47)	2.72	(0.83)	1.54	(0.43)	1.43	(0.45)						
*t*	0.68		1.41		0.19		0.27							

† *p* < 0.10.

* *p* < 0.05.

** *p* < 0.01.

*** *p* < 0.001.

*N* = 44 (Speaker *N* = 22 vs. Listener *N* = 22; couple = 26 vs. stranger = 18). SBP, systolic blood pressure; DBP, diastolic blood pressure; HR, heart rate; NA, negative affect; *t*-test values are for testing the differences in either experiment role (speaker vs. listener) or experimental condition (couple vs. stranger) in each cardiovascular activity indicator and negative affect.

Participants were randomly assigned to one of the two experimental role conditions (speaker vs. listener) just before the preparation phase began. As shown in speaker-listener rows in Table 2, the main effect of experimental role was significant on SBP [*F*_(1,42)_ = 5.24, *p* = 0.027] and DBP [*F*_(1,42)_ = 15.16, *p* = 0.001], marginally significant on NA [*F*_(1,42)_ = 3.80, *p* = 0.058], but not significant on HR [*F*_(1,42)_ = 1.12, *p* = 0.296]. Participants in the speaker condition had higher blood pressure levels than those in the listener condition. The experimental role of curvilinear time interaction effects was significant in all three cardiovascular activity markers and self-reported negative affect (*p*s < 0.004). At the baseline (before participants were assigned to an experimental role), DBP was higher among participants in the speaker condition, which was not expected. At the preparation and/or stress task phases, the group differences were significant across all cardiovascular activity markers and negative affect. At the recovery phase, DBP remained higher among participants in the speaker condition. Results confirm that giving a speech about the stressful situation that pertains to close relationships and health issues of the partner creates greater physiological and psychological strains than does listening to the speech.

Participants were also randomly assigned to one of two experimental pairing conditions (paired with own partner vs. stranger). As shown in couple-stranger rows in Table 2, the main effect of experimental condition was nonsignificant on all three cardiovascular markers and negative affect [0.17 < *F*s_(1,42)_ < 2.48, 0.12 < *p*s < 0.68]. Overall, being paired with one’s own partner did not produce outcomes that differed from outcomes when being paired with a stranger. As shown in time-by-condition effects columns in Table 2, the experimental condition-by-time interaction effects were marginally significant for DBP (those paired with their own partners increased DBP more than those paired with a stranger while preparing for the stress task) and HR (linear pattern of increases in HR in the couple condition while non-significant linear pattern in the stranger condition). Results suggest that imagining being involved in a car accident is stressful regardless whether the other person involved is a romantic partner or a stranger.

### Individual differences in responses to STITCH task

As shown in Table 3, attachment security was negatively related to SBP at the baseline, which tended to remain at both the preparation and stress task phases. A similar effect was also noted in DBP, showing marginally significant linear effect of negative association of attachment security with DBP, which became stronger as the study phases proceeded. However, with regard to self-reported negative affect, attachment security was positively associated at the baseline only. Attachment avoidance was not related to any outcomes studied. Attachment anxiety expressed as worry was positively associated with HR at baseline, which association decreased as the study phases proceeded. Attachment anxiety expressed as a desire to merge was negatively related to DBP at the baseline only and negatively related to NA at the recovery phase only.

**Table 3:** Individual difference effects on cardiovascular activity indicators and negative affect.

	Study phases								Time effects by study phases							
	Baseline		Preparation		Stress task		Recovery		Overall		Linear		Quadratic		Cubic	
	*B*	*t*	*B*	*t*	*B*	*t*	*B*	*t*	*F*	*p*	*F*	*p*	*F*	*p*	*F*	*p*
**SBP**																
**Adult attachment**																
Security	−10.22	−2.01^*^	−14.23	−1.75^†^	−15.88	−1.78^†^	−10.90	−1.66	3.81	0.059	0.07	0.793	0.68	0.414	0.28	0.603
Avoidance	−4.72	−0.99	−11.10	−1.45	−12.54	−1.49	−5.96	−0.96	1.93	0.173	0.15	0.698	1.61	0.213	0.16	0.691
Anxiety-worry	−1.51	−0.53	−1.33	−0.29	−1.22	−0.24	0.29	0.08	0.07	0.799	0.50	0.485	0.05	0.828	0.10	0.751
Anxiety-merger	−2.81	−0.95	−5.30	−1.13	−6.08	−1.18	−5.61	−1.47	1.70	0.201	1.28	0.266	0.22	0.641	0.01	0.925
**Big five personality**																
Extraversion	−2.69	−1.47	−3.66	−1.15	−1.68	−0.47	−4.12	−1.70	1.48	0.233	0.17	0.686	0.11	0.746	6.90	0.013
Agreeableness	−2.57	−0.90	−4.41	−0.88	−2.38	−0.43	−3.62	−0.95	0.69	0.413	0.02	0.900	0.01	0.932	2.62	0.115
Conscientiousness	−0.60	−0.33	−2.33	−0.73	−2.01	−0.57	0.24	0.10	0.23	0.638	0.25	0.618	0.80	0.379	0.01	0.971
Neuroticism	−1.81	−1.03	−2.21	−0.72	−2.04	−0.60	−0.20	−0.09	0.42	0.520	0.84	0.367	0.27	0.609	0.17	0.685
Openness	0.74	0.36	1.54	0.43	0.97	0.25	1.34	0.49	0.17	0.684	0.04	0.849	0.01	0.932	0.54	0.467
**DBP**																
**Adult attachment**																
Security	0.57	0.17	−2.98	−0.67	−3.46	−0.72	−4.76	−1.23	0.48	0.494	3.87	0.057	0.22	0.640	0.46	0.502
Avoidance	0.71	0.22	−2.73	−0.65	−5.39	−1.19	−2.98	−0.82	0.52	0.477	3.04	0.090	1.70	0.201	0.64	0.430
Anxiety-worry	2.74	1.45	2.22	0.89	2.97	1.10	2.42	1.12	1.46	0.235	0.01	0.963	0.01	0.990	0.65	0.425
Anxiety-merger	−4.27	−2.17^*^	−3.73	−1.44	−4.44	−1.59	−3.63	−1.61	3.26	0.079	0.06	0.805	0.01	0.920	0.71	0.406
**Big five personality**																
Extraversion	−0.83	−0.58	0.20	0.11	1.09	0.53	0.80	0.49	0.04	0.849	2.96	0.095	0.52	0.476	0.21	0.652
Agreeableness	1.37	0.61	0.96	0.33	1.34	0.41	1.57	0.60	0.25	0.618	0.03	0.855	0.05	0.825	0.07	0.798
Conscientiousness	−0.72	−0.50	−0.70	−0.37	−0.39	−0.19	0.08	0.05	0.07	0.795	0.65	0.426	0.06	0.806	0.01	0.954
Neuroticism	0.97	0.70	0.82	0.45	0.72	0.36	0.22	0.14	0.18	0.671	0.52	0.477	0.04	0.846	0.04	0.837
Openness	0.37	0.23	1.18	0.56	0.13	0.06	0.12	0.07	0.06	0.809	0.23	0.638	0.16	0.690	1.27	0.268
**HR**																
**Adult attachment**																
Security	−4.26	−0.79	−3.52	−0.40	−1.33	−0.16	−0.45	−0.08	0.13	0.720	2.80	0.103	0.01	0.990	0.24	0.628
Avoidance	−6.09	−1.20	−9.22	−1.11	−7.47	−0.94	−2.97	−0.55	1.07	0.307	2.10	0.156	0.50	0.483	0.16	0.693
Anxiety-worry	6.18	2.05^*^	5.14	1.04	5.44	1.15	1.88	0.59	1.60	0.215	7.66	0.009	0.16	0.696	2.74	0.107
Anxiety-merger	−0.19	−0.06	−2.34	−0.46	−2.69	−0.55	1.71	0.51	0.05	0.820	1.28	0.266	0.98	0.330	0.83	0.370
**Big five personality**																
Extraversion	1.93	0.97	1.32	0.42	0.99	0.33	0.03	0.01	0.22	0.642	2.90	0.098	0.01	0.937	0.14	0.710
Agreeableness	6.62	2.12^*^	13.21	2.70^**^	12.77	2.75^**^	5.09	1.61	7.01	0.012	0.82	0.373	4.13	0.050	0.01	0.956
Conscientiousness	−0.96	−0.49	−2.56	−0.83	−2.44	−0.83	−0.06	−0.03	0.45	0.508	0.64	0.429	0.80	0.378	0.06	0.814
Neuroticism	2.17	1.14	3.30	1.10	3.46	1.22	1.68	0.86	1.48	0.233	0.15	0.700	0.46	0.503	0.19	0.668
Openness	−3.48	−1.57	−3.89	−1.12	−4.14	−1.26	−4.37	−1.94^†^	2.46	0.126	0.54	0.468	0.01	0.972	0.01	0.965
**NA**																
**Adult attachment**																
Security	0.44	1.99^*^	0.14	0.23	0.45	1.28	0.42	1.66	2.01	0.165	0.05	0.821	0.12	0.728	0.43	0.516
Avoidance	0.32	1.53	0.01	0.02	0.26	0.78	0.16	0.67	0.60	0.443	0.04	0.838	0.08	0.780	0.42	0.520
Anxiety-worry	0.02	0.15	0.54	1.64	0.30	1.55	0.23	1.62	3.67	0.064	0.38	0.541	1.88	0.179	1.28	0.266
Anxiety-merger	−0.03	−0.27	−0.53	−1.56	−0.30	−1.48	−0.30	−2.60^*^	3.92	0.056	0.74	0.394	1.21	0.279	1.32	0.259
**Big five personality**																
Extraversion	−0.04	−0.50	0.09	0.36	0.04	0.29	−0.02	−0.17	0.03	0.859	0.01	0.970	0.33	0.567	0.08	0.786
Agreeableness	−0.18	−1.49	0.32	0.87	0.04	0.16	−0.10	−0.63	0.02	0.899	0.01	0.931	1.65	0.209	1.15	0.291
Conscientiousness	0.10	1.38	−0.19	−0.82	−0.07	−0.52	−0.12	−1.24	0.50	0.486	1.72	0.199	0.61	0.441	1.12	0.299
Neuroticism	0.23	3.15^**^	0.31	1.35	0.24	1.72^†^	0.16	1.65	5.52	0.025	0.44	0.514	0.26	0.612	0.07	0.791
Openness	−0.02	−0.18	−0.16	−0.63	0.20	1.27	0.12	1.06	0.10	0.759	2.54	0.120	0.04	0.852	2.44	0.128

† *p* < 0.10.

* *p* < 0.05.

** *p* < 0.01.

*** *p* < 0.001.

*N* = 44 (Speaker *N* = 22 vs. Listener *N* = 22; couple = 26 vs. stranger = 18). SBP, systolic blood pressure; DBP, diastolic blood pressure; HR, heart rate; NA, negative affect.

Among the five major personality factors, extraversion was marginally increased its positive association with DBP and decreased its association with HR as the study phases proceeded. Agreeableness was positively related to HR, which magnitude of association increased from the baseline through stress task phases, then became non-significantly associated at the recovery phase. Conscientiousness was not related to any study outcomes. Neuroticism was positively related to negative affect at the baseline and marginally so at the stress task phase. Finally, openness was marginally negatively related to HR at the recovery phase only. Results suggest cardiovascular and self-reported reactivity to the STITCH task tended to vary by individual characteristics that reflect one’s sensitivity to close relationship-and health-related stress.

## Discussion

The stress a medical illness imposes on the family of the patient and the impact of an illness diagnosis on quality of life and health outcomes can be substantial (Kim and Given, 2008; Kent et al., 2016). There likely are important individual differences in vulnerability to such stresses. However, proper tools to assess reactions to such types of stresses do not exist. Accordingly, we attempted to develop a task to induce stress in the laboratory, using an analog procedure that raises concerns similar to those that naturally arise in the close relationship and health context. Using a laboratory analog in which the same context is presented to everyone, rather than trying to assess stress regulation in the medical setting itself has several benefits. The task, which we call the STITCH task, required participants to visualize either their own romantic partner or a stranger being hurt by a hit-and-run car accident (a proxy situation of a medical illness in the family), or to visualize being the victim of such an accident.

This task proved to induce stress successfully, as evidenced in both cardiovascular activities and self-reported negative affect. Stress induced by the STITCH task provoked empathic cardiovascular and self-reported affect reactivities equally in romantic couples and pairs of strangers. As expected, reactivity was greater among those who were required to speak about the event than among those who had a more passive role. The results from this initial validation study provide sound evidence that the STITCH task can be a useful tool to induce close relationship and health-related stress. The STITCH task could be used to assess the extent to which the patient and family caregiver(s) are able to mutually use each other as resources for stress management. This, in turn, could be used as a predictor of later health outcomes.

Future studies will be fruitful in providing further validation of this newly developed stress induction tool. A particularly interesting direction for further work will be to examine phenomena predicted by relationship theories. For example, some have posited coregulation, in which two members of a dyad mutually calm each other’s reactions to a mutual stress (Butler, 2017; Randall et al., 2021). Elucidating the dyadic regulatory processes involving reactivity to and recovery from such the mutual stressors will help guiding precise and effective management of cardiovascular responses and negative affect for both members in the dyad. Identifying further individual and dyadic differences that moderate physiological and self-reported affective reactions to the STITCH task is also needed.

Validating the STITCH task with populations of medical patients and their caregivers as well as testing discriminant validity will be another important step. Furthermore, only the participants who are assigned to the caregiver role speak in the current STITCH task. Because public speaking *per se* is a psychological and physiological stressor, adding a phase where those assigned to the patient role also speak would help elucidate the effects of patients vs. caregivers from those of listeners vs. speakers. Finally, we hope to use this task to examine long-term implications of physiological and affective reactivities to the STITCH task in the quality of life and health outcomes of those who had experienced a medically stressful circumstance personally or in the family. Such information will expand current knowledge in understanding the impact of medical illness in the family and shed light on ways in which preventing premature aging and improving public health.

## Data availability statement

The raw data supporting the conclusions of this article will be made available by the authors, without undue reservation.

## Ethics statement

The studies involving human participants were reviewed and approved by the University of Miami. The patients/participants provided their written informed consent to participate in this study.

## Author contributions

YK developed the study concept and contributed to the conceptualization of the research goals and aims, data collection and analysis, and writing and reviewing of the manuscript. CC contributed to the conceptualization of the research goals and aims and writing and reviewing of the manuscript. BH contributed to the conceptualization of the research goals and data collection. All authors contributed to the article and approved the submitted version.

## Funding

The writing of this manuscript was supported by the National Institute of Nursing Research (R01NR016838) to YK.

## Conflict of interest

The authors declare that the research was conducted in the absence of any commercial or financial relationships that could be construed as a potential conflict of interest.

## Publisher’s note

All claims expressed in this article are solely those of the authors and do not necessarily represent those of their affiliated organizations, or those of the publisher, the editors and the reviewers. Any product that may be evaluated in this article, or claim that may be made by its manufacturer, is not guaranteed or endorsed by the publisher.

## Appendix

STITCH Scenario (Individualized with “Chris” = Survivor; “Robin” = Caregiver).

**Table:**

Instruction: Individualize the scenario before the session by substituting the patient and caregiver’s actual names into the script. Instruct participants to close their eyes and imagine as vividly as possible that they are experiencing the events you are about to describe. Read the scenario with emphasis on words that are in capitals, slowing down for phrases with embedded spaces, and louder for phrases and sentences in larger font.
*It was the birthday of a mutual friend of you, Chris, and you, Robin.* *The weather was just P E R f ect, so you decide to WALK to the friend’s birthday party.* *Your friend lives in a house i n a v e r y q u i e t residential area,* *where there are not many houses around.* *You, Chris and Robin, got the r e a r o und 7 p.m.* *The walk took ab o u t 30 minutes from your house.* *But walking was v e ry pLe asant, a n d r e l a xing.* *At the party, both of you had a G R r e at time catching up with many friends….* *It w a s around midnight, and both of you were leaving the party. You were the last people* *to leave….…. It was a b out, 15 minutes after you were walking back from the party,* *which is ha l f way home. O n c e again, v e r y p l e asant.*

*You, Robin,, See A Car Approaching; AND, Suddenly you realize.* *that the car is OUT OF control.* Leaving no time to react: the car **slams** into the sidewalk, missing you, Robin but **HItting** Chris. The driver stumbles out of the car, H ardly able to walk, approaching you and Chris. You R e alize that the driver is under the influence of alcohol. As the driver sees Chris **BLe e ding** on the ground, the driver gets Back in the car and Drives aWay, leaving you, and Chris HElpless in the middle of the road.

*You reach for your phone and* realize that you have LEFT it at the party; *you try to reach for Chris’ phone* and you realize that it has **N O** battery. **T h e r e is N O o n e a r o u n d**, and the neighborhood is **N O T** f a m i l i a r to you. Chris is bleeding from a wound and unconscious on the pavement. You **Y E L L** for help, and **n o o n e a n s w e r s**…. Chris is unresponsive. You beGin to P A nic, knowing that you need to get Chris to a hospital **immEdiately**…”
